# Recent Design Principles and Construction Strategies of Polyaniline-Based Composites: Toward Electrochemical and Non-Electrochemical Adsorption Applications

**DOI:** 10.3390/polym17233151

**Published:** 2025-11-27

**Authors:** Quanfeng Liang, Xu Wu, Yanghao Yan, Shaomin Kang, Jingjing Liu, Mingxing Shi, Guolin Tong

**Affiliations:** 1 College of Light Industry and Food Engineering, Nanjing Forestry University, Nanjing 210037, China; 2 College of Environment and Energy, Zhejiang Guangsha Vocational and Technical University of Construction, Jinhua 322100, China; 3 School of Chemistry and Chemical Engineering, Nanjing University of Science and Technology, Nanjing 210094, China

**Keywords:** polyaniline, composite design, interface engineering, environmental remediation, electrochemical adsorption

## Abstract

Presently, with rapid industrialization progressing, massive toxic pollutants have been discharged into nature, causing serious threats to ecosystems and human health. Thus, exploiting advanced functional materials for mitigating environmental challenges is vital. Among them, polyaniline (PANI)-based composites have gained great research attention because of their excellent mass–charge transfer ability, tunable morphology, rich N-containing functional groups, and high structural tunability. Herein, this review systematically summarizes the design principles, composite construction strategies, and adsorption applications of PANI-based composites. Key design principles, including micro-support skeleton construction, conductive skeleton introduction, and selective active site anchoring, are proposed. These principles aim to address defects of single components and realize the improvement of the properties, selectivity, and stability of PANI-based composites. Subsequently, multiple advanced PANI-based composites are further analyzed. Eventually, their applications in electrochemical (e.g., electrosorption) and non-electrochemical adsorption (e.g., typical adsorption) are comprehensively assessed. Overall, this review seeks to deliver valuable insights into the in-depth study of advanced PANI-based composites for effective pollutant remediation.

## 1. Introduction

Currently, accelerated global industrialization and urbanization have caused serious environmental crises that affect water, air, and soil [1,2]. Therefore, it is vital to explore effective technologies for deep pollutant remediation. Considering the different characteristics of diverse pollutants, multiple strategies have been successively developed, such as photocatalysis, peroxymonosulfate (PMS) activation, membrane methods, and electro-Fenton processes [3,4,5]. However, they often face high costs, short service life, serious membrane fouling, and the generation of massive toxic byproducts [6]. In contrast, adsorption, including electrochemical and non-electrochemical adsorption, has now attracted huge research interest. The former refers to electrosorption, i.e., capacitive deionization (CDI), which is low-cost, completely pollution-free, easy to operate, and highly scalable [7], while the latter is a typical adsorption method with similar advantages [8].

Adsorption materials are the core of adsorption techniques, which greatly impact the adsorption capacity, adsorption dynamics, and cycle stability [9,10]. Orthodox adsorption materials face inherent limitations. For instance, porous carbons (PCs) lack wide ion applicability and have few selective active sites, while metal oxides suffer from poor conductivity and porosity. Metal–organic frameworks (MOFs) have low water stability and easily agglomerate, while covalent organic frameworks (COFs) are costly to synthesize [11,12,13]. These drawbacks highlight the requirement for synergistic composite materials that integrate multiple functions. Polymer-based composites, especially those with conductive polymers (e.g., polyaniline (PANI), polypyrrole (PPy), and polythiophene (PTh)), have gained attention ascribed to their tunable molecular structures and interface compatibility [14,15,16]. Among these, PANI stands out for its intrinsic conductivity, plentiful functional groups, low-cost synthesis, and good compatibility with other components, making it not only a promising solution for environmental remediation but also suitable for sensors and energy storage and conversion [17,18,19,20,21,22].

The structural advantages of PANI integrated with other materials depend on its unique “bridge effect”, i.e., material joint function. This effect can achieve the effective introduction or integration of customized morphology structure, conductive network, and surface functional groups [23,24,25]. For porous supports that have a large specific surface area (S_BET_) and pore volume (V_pore_), like carbon aerogels (CAs) and MOFs, PANI with a positively charged backbone reasonably fills pores, coats surfaces, or separates particles to inhibit agglomeration while introducing plentiful amine and imine groups [26,27]. For conductive components such as MXene and metallic oxide, PANI establishes continuous conductive networks to reduce charge transfer resistance [28,29]. Notably, the performance of PANI is highly dependent on its molecular regulation, e.g., polymerization method and doping degree, which directly impact composite interface quality, exposed accessible active sites, and ultimate functionality [30]. For example, chemically polymerized PANI with excellent dispersibility is more suitable for typical adsorption, while electrochemically polymerized PANI with regular chains is preferred for electrosorption [31,32].

**Figure 1:**
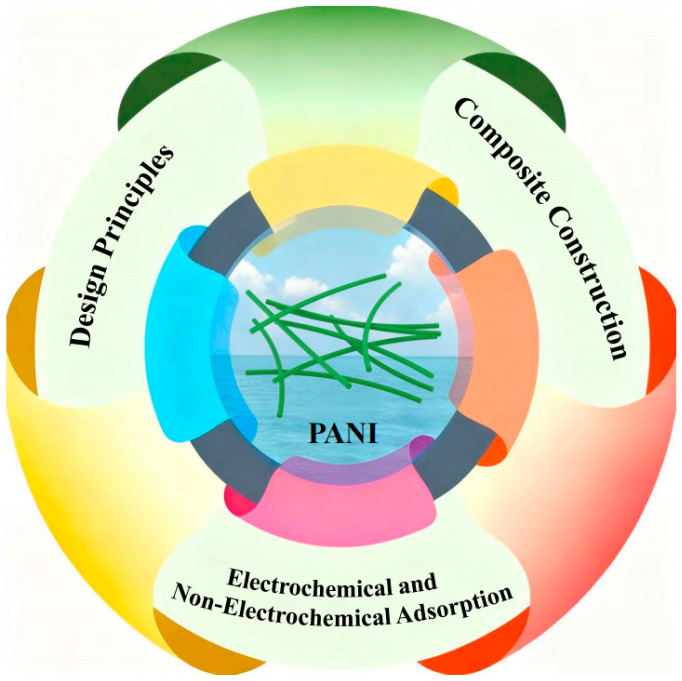
Schematic representation of the design principles, construction strategies, and adsorption applications of PANI-based composites.

Herein, PANI-based composites for effective adsorption applications are analyzed due to their tunable morphology, rich functional groups, superior conductivity, and high structural tunability. Specifically, this review comprehensively overviews the design principles, composite construction strategies, and adsorption applications (Figure 1). Firstly, key design principles, e.g., micro-support skeleton construction, conductive skeleton introduction, and selective active site anchoring, are proposed to optimize the property, selectivity, and stability of PANI-based composites. Subsequently, multiple PANI-based composites are further analyzed. Eventually, their environmental applications in electrochemical and non-electrochemical adsorption are systemically appraised, involving desalination, toxic anion/heavy metal ion removal, organic pollutant/harmful gas adsorption, water softening, and resource recovery.

## 2. Intrinsic Properties and Polymerization Regulation of PANI

### 2.1. Molecular Structure and Intrinsic Performance of PANI

PANI, fabricated by chemical or electrochemical polymerization of aniline, is one of the most researched conducting polymers. Normally, PANI holds three molecular structures, involving the fully reduced leucoemeraldine base (LEB), half-oxidized emeraldine base (EB), and fully oxidized pernigraniline base (PAB) [33]. Notably, the emeraldine salt (ES) form, acquired via EB protonation, is the most widely investigated owing to its balanced conductivity and high chemical stability [34]. Among these forms, its adjustable conductivity can be realized by doping the ES with protonic acid. Its molecular chain contains alternating diamine and diimine units, with conjugated π-electrons enabling rapid charge transfer, and surface N-containing functional groups act as adsorption sites via hydrogen bonding or coordination bonding [35,36]. Notably, the key intrinsic characteristics of PANI relevant to adsorption applications include adjustable structures that can maximize surface accessibility, high surface reactivity where positively charged surface and copious amine and imine groups interacts with pollutants, excellent conductivity that determines performance in enhanced adsorption and electrosorption, and satisfactory stability that resists acid/alkali corrosion but is prone to oxidation in strong oxidizing environments, requiring composite modification.

### 2.2. Molecular-Level Modification of PANI

Molecular modification of PANI can further optimize the performance. Orthodox molecular modification involves proton doping and functional group grafting [37,38,39]. EB-type PANI delivers unique proton conductivity, with efficient proton hopping occurring across hydrogen-bonded networks. In the hydrated state, H_2_O molecules adsorbed on the PANI surface dissociate into H^+^ and OH^−^. Protonated imine nitrogen sites in the PANI backbone serve as proton-hopping locations, and proton transfer proceeds via the Grotthuss mechanism, i.e., protons migrate along hydrogen-bonded water chains [40,41]. Research illustrated that proton doping with HCl or H_2_SO_4_ can effectively increase the number of charge carriers in PANI chains and improve the conductivity, and doped PANI is more compatible with conductive components because of reinforced electron delocalization [42,43]. Meanwhile, functional group grafting of -COOH, -SO_3_H, or -SH onto PANI chains can strengthen coordination with metal ions [37,38,39].

### 2.3. Polymerization Regulation of PANI and Its Impact on Composite Performance

The polymerization method of PANI directly impacts the molecular weight, crystalline/morphology structure, and particle dispersion, being vital for composite compatibility and functionalization. For morphologies, PANI has multi-structures involving nanoparticles, nanosheets, nanotubes, nanowires, nanofibers, and nanorods [22,44,45]. In polymerization methods, chemical oxidative polymerization adopts oxidants like FeCl_3_ or (NH_4_)_2_SO_4_, inducing PANI with a broad molecular weight distribution and high dispersibility, making it suitable for supports to reasonably fill pores, coat surfaces, or separate particles [46]. Electrochemical polymerization utilizes a three-electrode system, producing PANI with regular chain arrangement, high crystallinity, and controllable thickness, favoring electrosorption, e.g., PANI/activated carbon fiber (ACF) [47] and PANI/GP [48]. Moreover, there are still other advanced synthesis methods, e.g., interfacial polymerization, plasma polymerization, and sonochemical synthesis [49,50]. Overall, due to the diverse morphologies of PANI, the rational design core of PANI-based composites for electrochemical and non-electrochemical adsorption is crucial.

Overall, viewing the above three characteristics of single PANI, it shows great promise for developing PANI-based composites for adsorption applications. However, the purpose of adopting reasonable design principles to build composites with effective 3D structures with highly exposed active sites (e.g., porosity and functional groups) is vital, as this is core to enhancing the performance of composites.

## 3. Design Principles of PANI-Based Composites for Adsorption

### 3.1. Micro-Support Skeleton Construction

Micro-support skeleton construction is a vital manufacturing principle for PANI-based composites, with a universal focus on surface accessibility, mass-transfer ability, and structural stability. This is well adapted to wide adsorption applications. Notably, it mainly provides a robust framework for porous materials (e.g., PCs, MOFs, and COFs) that are apt to aggregation because of their multiple and well-designed structures, like tube-type structures [26,27]. Specifically, maintaining an intrinsic or creating a hierarchical porous structures favors electrolyte permeation and ions/molecules diffusion (macropores and mesopores), along with maximizing micropore accessibility of composites [51]. This provides a powerful material platform for subsequent adsorption processes. Moreover, the “bridge effect” from PANI and the subsequent design of interconnected 3D porous structures can greatly reinforce composite conductivity, ion diffusion dynamics, and structural stability [52]. Notably, the micro-support skeleton effect is mutual, which is ascribed to the fact that PANI also exhibits easily aggregated spheres or sheets [53]. Overall, the synergistic effect between PANI and other materials constituting the microscopic skeleton ensures optimized interfacial compatibility, high and rapid surface-site accessibility, and improved structural stability.

### 3.2. Conductive Skeleton Introduction

Conductive skeleton introduction is important for conduction continuity and mass/charge transfer in PANI-based composites. Its core goal is to strengthen conductivity and maximize active site utilization of the composite, while tailoring to the demands of CDI. From the perspective of composite structure, there often have four types of conductive modes: (i) hybridization of PANI nanosheets with other nanoparticles, e.g., massive small nanoparticles uniformly dispersed on the surface of PANI nanosheets (e.g., Mo_4_/_3_CT_z_/PANI [54]); (ii) hybridization of PANI nanotubes/nanowires/nanofibers/nanorods with other nanoparticles, e.g., PANI with a high aspect ratio and intertwining forming continuous conductive frameworks and nanoparticles uniformly dispersed on its surface (e.g., P-PANI/AC [55]); (iii) hybridization of PANI nanotubes/nanowires/nanofibers/nanorods with other nanosheets (e.g., Rh/PANI-MX [56], PANI-Cl_x_/Ti_3_C_2_T_x_ [57]), which requires PANI to intertwine with each other and be pillared between nanosheets; and (iv) core–shell structure or cladding structure, e.g., PANI coated on the surface of other materials (e.g., Fe_3_O_4_@PANI [58], MWCNTs@Co/C@PANI [59]). Overall, these structures can realize rapid mass/charge transfer and the performance release of active sites. If CDI is coupled with other techniques such as peroxymonosulfate activation or photocatalysis, it can also favor electron transfer between composites, pollutants, and PMS, or the separation and utilization of photogenerated carriers, thus enhancing pollutant-removal properties [60,61,62].

For traditional adsorption applications, conductivity itself does not directly determine the adsorption capacity, but it can improve the adsorption property in the following ways [63]. Firstly, for the adsorption of some charged pollutants (e.g., heavy metal ions and organic anions), material conductivity means that there are movable charge carriers which can adjust the surface potential and enhance electrostatic interactions [64]. Secondly, composites with excellent conductivity usually have better charge-transport ability, which may promote the adsorption kinetics of pollutants on the composite surface, such as faster migration of ions in the conductive network and reduced diffusion resistance [65].

### 3.3. Selective Active Site Anchoring

PANI is both a “basic function provider” and a “customizable platform” [37,38,39]. For the former, PANI, with its positive charge, holds plentiful amine and imine groups, which are more favorable for the adsorption of organic anions. Meanwhile, it can provide extra pseudocapacitor and deliver higher anion adsorption selectivity and capacity when taken as an electrochemical adsorption electrode. For the latter, the introduction of other functional groups (e.g., -COOH, -SO_3_H, or -SH) can break through its inherent selectivity limitations and meet the needs of precise capture of target substances in different scenarios. For instance, -COOH-functionalized PANI/halloysite displayed excellent adsorption ability to uranium, while -PO_4_^3−^-functionalized PANI/AC acquired superior thorium-selective electrosorption. The desalination mechanism of the latter is attributed to P=O → Th and >N− → Th [55]. In another research, Li et al. [66] adopted chemical oxidation polymerization to grow PANI in situ on PCs with meso-macroporous channels, which realized good dispersibility of PANI and reduced pore blockage of PCs, retaining 85% of the original S_BET_.

## 4. PANI-Based Composite Construction: Structure-Performance Correlation

### 4.1. Polyaniline/Porous Carbon Composite

PCs have high S_BET_, large V_pore_, adjustable pore-size distribution, and excellent structural stability [67,68]. Common PCs involve graphene, activated carbons (ACs), heteroatom-doped carbons, biomass-derived carbons, etc. [69,70]. The composite principle between PANI and PCs lies in their synergistic effects in structure, interface, and electron conduction. Specifically, PCs with copious hierarchical porosity provide a stable supporting framework for PANI. This prevents the agglomeration of PANI, thus improving surface accessibility and optimizing mass-transfer ability. Meanwhile, PCs can manufacture efficient conductive pathways depending on their excellent graphitized network [71]. In contrast, PANI offers plentiful surface functional groups, including amine and imine groups. PANI coupled with PCs can further improve interfacial stability and electron transfer ability by hydrogen bonding and π-π conjugation [72]. This design remedies the inherent defects of individual materials, e.g., low S_BET_ and poor cyclic stability for PANI, and strong surface inertness and single EDLC capacitance for PCs [73].

Notably, common PCs often have massive micropores. Although this can hinder the aggregation of PANI, it greatly blocks the pores of PCs. Thus, it is vital to realize the effective utilization of the inner space of composites and improve diffusion kinetics. Li et al. [66] exploited unique porous ACs (PACs) with adjustable meso-macroporous channels based on a tableting-pressurization method. Then, PAC/PAN was fabricated by an in situ growth method. Notably, PANI with thin thickness was only loaded on the surface of PACs rather than into their interior. As a result, the well-designed structure retained rapid ion-transport channels and effectively couples EDL capacitance with pseudocapacitance. To further accelerate the adsorption rate, Wang et al. [74] prepared a PANI@nano-hollow carbon sphere. Research indicated that the hollow structure of hollow carbon spheres could be well-maintained by effectively controlling the loaded PANI thickness at about 90 nm; otherwise, it will be fully covered by PANI. Thus, the unique hollow composite structures effectively accelerated the diffusion, adsorption, and reduction in Cr(IV), and the adsorption capacity was 190.8 mg g^−1^ in 100 mg L^−1^ Cr(IV) at pH = 1.

Structural uniformity directly impacts the electron- and ion-transport behavior in composites. To achieve uniform assembly of PANI on PCs, Sun et al. [75] adopted lignin (Lig) as a molecular modulator to build a homogeneous structural PANI/Lig/GO sheet by π-π interaction and hydrogen bonding. Further, a 3D porous PANI/Lig/RGO hydrogel/CC (PLRH/CC) was constructed by carbothermal reduction of PANI/Lig/GO@carbon cloth (CC). SEM data indicated that the introduction of Lig could realize the uniform assembly of PANI between the RGO layers. In contrast, the PANI/RGO hydrogel/CC showed a stacked structure and reduced surface accessibility. The optimal PLRH/CC delivered a high S_BET_ of 74.45 m^2^ g^−1^, large V_pore_ of 0.35 cm^3^ g^−1^, and superior disorder degree of I_D_/I_G_ = 1.66. Overall, the combination of the S_BET_ of PCs and the polar sites of PANI achieves high adsorption performance, while the support effect of PCs inhibits the volume expansion of PANI, strengthening the cyclic stability of PANI/PC composites.

### 4.2. Polyaniline/Metallic Oxide Composite

Metal oxides have controllable morphologies, plentiful active sites, certain adsorption selectivity, high stability and durability, which make them suitable for both adsorption and catalysis. Typical metal oxides include TiO_2_, ZnO, Fe_3_O_4_, WO_3_, ZrO_2_, etc. [72,76,77]. Based on their adsorption and catalysis mechanisms, they can effectively capture various pollutants or degrade toxic pollutants into low-toxic byproducts. However, multiple problems, such as particle aggregation, toxicity issues, and cost-effectiveness, are inevitable [78]. Metal oxides provide support for PANI to inhibit agglomeration because of their regular morphology and plentiful metal active sites, while PANI heightens the conductivity of metal oxides via its conjugated structures. The composite combination makes up for the problems of poor conductivity and weak cycling stability of metal oxides and the low S_BET_ and insufficient adsorption selectivity of PANI [79]. Thus, in adsorption, metal oxides selectively capture heavy metals/anionic pollutants, and the amine and imine groups of PANI assist in adsorption and stabilize the structure [80]. In CDI, PANI improves conductivity and capacity, while metal oxides inhibit the volume expansion of PANI to maintain cycling performance [81].

Fe_3_O_4_ has been widely applied in adsorption due to its inherent magnetic properties. This trait enables efficient separation of Fe_3_O_4_ from water via magnetic fields after the adsorption completion, thus ensuring its reusability. Wang et al. [77] developed unique PANI@Fe_3_O_4_ nanorods (PANI@Fe_3_O_4_). Then, multiple DESs-based magnetic nanomicrospheres (DESs-PANI@Fe_3_O_4_) were fabricated by grafting diverse deep eutectic solvents (DESs). SEM data indicated that the optimal composite depicted spherical micro-nanoporous structures with Fe_3_O_4_ as the core–shell, with a large S_BET_ of 65.24 m^2^ g, high N doping level of 10.9%, and copious –OH, –NH, and –COOH groups. When used for the capture of methyl orange (MO) and methylene blue (MB), it achieved high adsorption capacities of 812 mg g^−1^ and 446 mg g^−1^ in a 300 mg L^−1^ solution within 130 min, respectively. Their adsorption behaviors were mainly controlled by π-π interaction, hydrogen bonding, and van der Waals interaction. Analogously, Goswami et al. [82] adopted PANI@Fe_3_O_4_ for 2, 4-dichlorophenoxyacetic acid capture, reaching a high adsorption capacity of around 28.5 mg g^−1^ in a 100 mg L^−1^ solution within 100 min.

TiO_2_ holds improved hydrophilicity and excellent pseudocapacitance, and it normally has diverse shapes such as hollow spheres and tubes. Zhao et al. [83] developed a PANI-modified, highly ordered TiO_2_ nanotube array (EC-PANI/TiO_2_ NTA) via electrochemical polymerization. SEM data indicated that single TiO_2_ held thin tube walls and wide inner diameters of about 5–10 nm and 70–100 nm, respectively. By controlling the concentration of aniline as low as 0.1 M, the hollow structure of PANI/TiO_2_ NTA could be well exposed. Further characterization data displayed that it owned a high N-doped level of 12.98% and acquired higher catalytic ability. Thus, it realized a superior degradation efficiency of 94.3% and excellent 10th-cycle stability for 10 mg L^−1^ tetrabromobisphenol A. In another research, Bian et al. [84] designed a PANI-TiO_2_ and realized the 100% deep reduction of Cr(VI) to Cr(III) by varying the loading of PANI on the mesoporous TiO_2_ surface. Additionally, Wang et al. [85] fabricated a partially carbonized core–shell Fe_3_O_4_@PANI-p with highly exposed accessible surface via in situ polymerization coupled with controlled pyrolysis for adsorption and subsequent PMS activation of tetracycline (TC). Mansoor et al. [86] gained a ZnO-based N-doped GO with PANI (ZnO/N-GO/PANI) with uniformly dispersed spherical and lamellar particles, a high S_BET_ of 31.65 m^2^ g^−1^, and excellent specific capacitance of 628.4 F g^−1^. When adopted for enhanced flow CDI (FCDI) desalination, realizing a high salt adsorption capacity of 119 mg g^−1^ in 1000 mg L^−1^ NaCl at 1.2 V. Viewing the diverse characteristics of metal oxides, their application potential in specific environmental fields tends to be distinguished, needing further reasonable design.

### 4.3. Polyaniline/MXene Composite

MXenes, a compelling family of 2D materials, possess a general chemical formula of Mn_+1_X_n_T_x_. Thereinto, M denotes transition metals (e.g., Ti, Mo, Ti, etc.), X stands for C or N, n refers to the layer numbers, and T represents functional groups (e.g., -OH and -F) [87]. Considering their unique layered structures, excellent conductivity, and variegated chemical properties, Mxenes have depicted huge potential in sensing detection, environmental remediation, and energy storage [88,89,90]. MXene, with unique layered structures, high conductivity, and copious -OH and -F groups, provides a stable substrate for PANI loading, which effectively inhibits the agglomeration, prevents interlayer stacking, and builds rapid electron transport pathways [91]. In turn, PANI supplements active sites and fills the interlayer gaps of MXene to increase S_BET_. Meanwhile, PANI and MXene can form strong interfacial interactions via hydrogen bonding and coordination bonding [92]. This favors alleviating serious interlayer stacking and poor adsorption selectivity of MXene, thus improving its agglomeration-sensitive conductivity and poor cycling stability.

Flow electrodes in FCDI are normally limited by discontinuous conductivity. To optimize the obstacle, Lu et al. [93] developed an F-selective MS-Ti_3_C_2_T_x_ MXene/PANI/AC (MTPA). Thereinto, PANI was in situ polymerized and evenly loaded on the AC surface. Subsequently, MXene was further tightly doped on the PANI/AC surface. Notably, the tight combination along with copious pores made MS-Ti_3_C_2_T_x_ MXene/PANI/AC a 3D connection structure with outstanding bridging and filling effects. Owing to its copious charge transfer routines, high disorder degree, and high specific capacitance of 132.4 F g^−1^, MTPA realized a rapid average removal rate of 3.415 mg m^−2^ s^−1^ in 10 mg L^−1^ NaF at 1.2 V, which was separately 1.72 and 1.37 times that of single AC and PANI/AC, respectively.

The positive charge of PANI is a powerful composite substrate to neutralize the over-negative charge of other materials. Based on electrostatic self-assembly, Sun et al. [92] exploited a unique PANI/MXene/GO (PMG) with irregular spherical nanohybrids, where MXene presented disorder ultrathin nanosheets with a thickness of 1–2 nm. Notably, the produced evenly dispersed PANI induced MXene and GO to intercalate with each other, thus ultimately relieving the over-negative potential of MXene/GO. Due to its high S_BET_ of 15 m^2^ g^−1^, reasonable average pore size of 20.39 nm, and strong mass/charge ability, PMG acquired a high adsorption capacity of 156.2 mg g^−1^ for Cr(VI) at pH = 2 in 80 mg L^−1^ Cr(VI). Overall, the layered structures of MXene and the collaborative effect of the active sites of the two components selectively adsorb heavy metals/polar organics. Meanwhile, the EDL capacitance from MXene combined with the pseudocapacitance from PANI can greatly enhance total specific capacitance.

### 4.4. Polyaniline/MOF Composite

MOFs, a category of porous materials manufactured by metal ions (such as Zn^2+^, Al^3+^, Co^2+^) or clusters linked to organic ligands via coordination bonds, generally have high S_BET_, large V_pore_, adjustable morphologies, and customizable pore sizes [94]. The structural diversity of MOFs makes them exhibit broad practicability in adsorption, catalysis, and drug loading. However, when applied specifically for liquid-phase adsorption, single MOFs often face inadequate stability, low adsorption rate, and narrow pH response ranges [95]. The strategy of combining PANI and MOFs is an effective way to alleviate the above shackles. Normally, MOFs can provide a uniform supporting matrix for PANI to inhibit agglomeration. Meanwhile, due to the copious porosity and metal sites, MOFs endow the composites with high adsorption potential [96]. In contrast, PANI greatly enhances the conductivity of MOFs via its conjugated π-bond structures. Its surface amino and imino groups can also shape coordinate bonds and hydrogen bonds with the metal ions or ligand functional groups of MOFs, which strengthens the interfacial bonding strength [97]. Therefore, PANI/MOF composites may address the collapse tendency in aqueous environments and weak cycling stability of MOFs, along with their low S_BET_, and further optimize the adsorption selectivity of PANI.

Developing waste plastic-derived PANI/MOF is a sustainable composite production craft. For instance, Kim et al. [98] utilized polyethylene terephthalate bottles as precursors to prepare an Fe-MOF@PANI. SEM data revealed that Fe-MOF@PANI held highly dispersed spindle-shaped structures with high surface accessibility, where PANI particles were evenly loaded on the surface of spindle-shaped Fe-MOF. Meanwhile, it possessed a high S_BET_ of 73.17 m^2^ g^−1^, wide pore-size distribution of 3–10 nm, and plentiful N/O functional groups. When taken for the capture of Cd(II) and Pb(II), it realized outstanding capture capacities of 143.36 and 258.59 mg g^−1^ in a 500 mg L^−1^ solution at pH = 6, respectively. Mechanism analysis disclosed that the adsorption of Cd(II) and Pb(II) on Fe-MOF@PANI was chemical adsorption-dominated monolayer adsorption. To reach separation and recycling of powder materials, Sahu et al. [99] designed a self-supported UiO-67-PANI-modified multilayer membrane. Thereinto, PANI provided a substantial frame and made the composite loose, effectively improving the MOF layer stability. Thus, it promoted the ultrafiltration membrane to a loose nanofiltration membrane, acquiring a 99.7% adsorption rate of Congo red while keeping nearly 100% salt passage in 1000 mg L^−1^ NaCl.

Building interpenetrating structures, such as extending PANI chains into the pores of MOFs, can greatly increase the utilization of the inner S_BET_ of MOFs and maximize composite conductivity. However, the design is difficult because the narrow pore sizes induce insufficient aniline amount. Innovatively, Chi et al. [100] developed a hollow hierarchical Cr-Al-MIL-101 (HH-MIL-101) based on the selective etching of its core and Al metal zones. Then, 3D interpenetrated HH-MIL-101/PANI was fabricated by electrochemically polymerizing PANI chains inside the MOF skeleton. As a result, HH-MIL-101/PANI possessed ultrahigh surface accessibility and conductivity with a high S_BET_ of 12 m^2^ g^−1^, a reasonable average pore size of 5.95 nm, and a splendid specific capacitance of 1934 mF cm^−2^. Overall, exploring highly conductive self-supporting porous PANI/MOFs is a promising research direction.

### 4.5. Polyaniline/LDH Composite

Layered double hydroxide (LDHs), a category of inorganic compounds with distinctive layered structures, are also commonly termed anionic clays. LDHs exhibit extensive applicability across multiple fields, ranging from drug delivery to environmental remediation [101]. Chemically, LDHs are constituted by metal cations and anions. Their unique layered structure, abundant surface -OH groups, high structural tunability, and excellent stability endow them with multifunctional properties [102]. To achieve efficient adsorption and reinforce recyclability, LDHs can be subjected to modification or functionalization treatments, such as incorporating specific polymers or inorganic components [103]. LDHs provide a supporting matrix and enhance selectivity by their layered channels and metal sites, while PANI improves the electrical conductivity of LDHs through its conjugated structure [104]. The strong interfacial bonding between the two compensates for the poor electrical conductivity and easy agglomeration of LDHs and the small S_BET_ and single selectivity of PANI.

PANI/LDHs integrate LDHs’ anion-exchange capacity with PANI’s redox activity, effective in anion removal via multiple interactions. Xu et al. [105] adopted carbon spheres (CSs) as carriers, preparing a unique ternary core–shell CS@PANI@LDH that had the following advantages: (i) the CS core prevented the aggregation of PANI and LDHs, thus retaining more available active sites; (ii) PANI beteer combined LDHs with CS and made the core–shell structural composite more stable; (iii) the composite contained massive -NH_2_ groups, favoring the selective adsorption of anionic pollutants. As expected, it achieved a superior capture capacity for diclofenac sodium (DCF) of 618.16 mg g^−1^ in 500 mg L^−1^ within an ultrarapid 60 min. Further, the group [24] adopted ZIF-8-derived carbon (ZC) as carriers, building another core–shell structural ZC@PANI@NiAl-LDH with an excellent S_BET_ of 49.8 m^2^ g^−1^ and a large V_pore_ of 0.19 cm^3^ g^−1^ [24]. The adsorption mechanism between the composite and saccharin involved electrostatic interactions, π-π interactions, and hydrogen bonding.

Song et al. [29] fabricated a novel LDH@MOF/PANI, where PANI served as a conductive framework and Al-MOF acted as a sacrificial template for LDHs. SEM data disclosed that Al-MOF and LDH/MOF possessed 3D gear-like morphologies and layered structures, respectively. The structural transformation of LDH/MOF made the eventual LDH@MOF/PANI a continuously conductive, mesopore-dominated layered structure with surface evenly dispersed interconnected porous PANI. Moreover, viewing its certain S_BET_ of 10.04 m^2^ g^−1^ and large V_pore_ of 0.06 cm^3^ g^−1^, it exhibited obviously selective adsorption for phosphorus. Mechanistic research disclosed that the possible phosphorus capture mechanism mainly involved electric field interaction, ligand exchange, hydrogen bonding, electrostatic interaction, and anion exchange. Innovatively, to increase the adsorption energy of NH_4_^+^, Fu et al. [106] fabricated Ni/Co-LDH nanocages (HLDH@P) with unique hollow structures and PANI coating, where uniformly dispersed PANI provides abundant N-containing groups with NH_4_^+^ selectivity. Overall, LDHs selectively capture anionic/cationic pollutants, and N-containing functional groups of PANI assist in adsorption and enhance selectivity. Their structural complementarity greatly improves the structural stability and mass–charge transfer performance.

### 4.6. Polyaniline/PBAs Composite

Prussian blue analogs (PBAs), featuring unique 3D open-framework structures, have been widely explored in the energy and environment fields. This is primarily attributed to their tunable composition, controllable open structure, and high physicochemical stability [107]. The chemical formula of PBAs is A_x_M′_y_[M(CN)_6_]_1−y_·nH_2_O, in which A represents alkali metal cations and M/M′ denotes transition metal cations. Linear anions act as bridges between M and M′, thereby generating a face-centered cubic open-framework structure with A sites that have large interstitial spaces [108]. Notably, when M = M′ = Fe, it is Prussian blue (PB). Further, diverse PBAs can be obtained by varying the metal element types. This open structure can store alkali ions or small molecules, which meet the demand of adsorption materials and the insertion/extraction mechanism of CDI materials. For PANI/PBAs composite, PBAs supply support for PANI to inhibit its agglomeration via filling their open pores and multiple metal sites [109]. Meanwhile, they also hold certain ion-recognition capability. In contrast, PANI strengthens the conductivity of PBAs via its conjugated structure, and its amino groups can form coordinate bonds with the metal ions in PBAs to achieve a strong interfacial effect [110]. This synergistic effect effectively remedies the poor electrical conductivity and weak cycling stability of PBAs, along with a low S_BET_ and agglomeration tendency of PANI.

Moazezi et al. [111] exploited an advanced PANI/Co-PBA through a rapid co-precipitation method. SEM data indicated that single PANI exhibited capsule-like particles with sizes of ca. 75 × 175 nm, while PANI/Co-PBA presented polyhedral particles with sizes of 80–100 nm. Meanwhile, more pores between the particles were generated, thus achieving a high S_BET_ of 35.12 m^2^ g^−1^ and a superior V_pore_ of 24.71 cm^3^ g^−1^. Further, the adsorption experiments of Rb^+^ on PANI/Co-PBA displayed that evenly dispersed PBAs and highly exposed active sites of the composite played a dominant role. Notably, the irreversible structural collapse and sluggish diffusion dynamics of PBAs often limit their further employment. Innovatively, Liu et al. [110] developed multiple ultrafine core–shell structural Mn-PBA@polymers via in situ polymerization coupled with an encapsulation strategy inspired by theoretical calculations. Experiment data indicated that compared with Mn-PBA@Ppy and Mn-PBA@ poly(3,4-ethylenedioxythiophene), Mn-PBA@PANI possessed the highest adsorption capacity for NH_4_^+^. This outstanding performance is mainly attributed to the significant synergy between Mn-PBA and PANI, which significantly enhanced the diffusion kinetics of NH_4_^+^ and structural stability of the composites. Overall, the metal sites and open pores of PBAs can rapidly and selectively capture heavy metals and NH_4_^+^, while PANI assists in adsorption. Meanwhile, PANI can accelerate mass/charge transfer, and PBAs inhibit the volume expansion of PANI.

## 5. Electrochemical and Non-Electrochemical Adsorption Applications

### 5.1. Adsorption

Adsorption is the phenomenon in which ions or molecules are concentrated on the solid surface [112]. Different from ozonation, biodegradation, and membrane technology with high costs, limited durability, membrane fouling, and the generation of toxic byproducts, adsorption has garnered considerable interest due to its cost-effectiveness, operational simplicity, high efficiency, and eco-friendly nature [113]. Notably, in comparison with single materials such as clay (e.g., montmorillonite, kaolin, and LDHs), MOFs, COFs, and MXene, PANI-based composites have attracted impressive attention because of their diversified morphological structure, highly exposed accessible S_BET_, superior functional characteristics, improved hydrophilicity, and excellent stability [114]. They can not only adsorb ions (e.g., heavy metal ions and radionuclides) and organic molecules (e.g., pharmaceuticals and dyes) but also capture gases including CO_2_ and VOCs.

Table 1 systematically lists the adsorption performance of PANI-based composites toward diverse pollutants by associating key parameters (adsorbent, pollutant type, adsorption conditions) with adsorption performance. It is clear that PANI-based composites possess broad adsorption applicability and tunable selectivity. Specifically, when PANI is combined with porous materials (e.g., PCs, MOFs), the composite’s pore structure and active site density are enhanced, favoring the complexation adsorption of cationic heavy metals (e.g., Pb^2+^, Hg^2+^). When combined with anion-exchange materials (e.g., LDH), it leverages LDH’s layered channel characteristics and PANI’s protonated positive sites to strengthen the adsorption of anionic pollutants (e.g., Cr(VI)) and polar pharmaceuticals. Notably, compared to the adsorption of heavy metal ions, which requires a certain choice of materials, the vast majority of PANI-based composites are suitable for the adsorption of organic pollutants. As capacity, selectivity, stability, and regenerability continue to be optimized, PANI-based composites hold promise for ensuring efficient pollutant adsorption across a wide range of water sources.

#### 5.1.1. Heavy Metal Ions

Currently, there are many types of heavy metal ions in water systems, e.g., Cu(II), Ni(II), Cr(IV), Pb(II), and Cd(II) [124,125]. What is worrying is that their harm to water bodies possesses the characteristics of refractoriness, bioaccumulation, and multi-target toxicity even at low concentrations. The hazard will be transmitted along the ecological chain of the water body and ultimately threaten the ecosystem and human health [126,127]. Adsorption method has always been a widely used and effective technique for removing heavy metal ions because of its low expense, simple operation, universal adsorption range, and diverse adsorption mechanisms [128].

Via the hydrothermal reaction of Mg/Al LDH and in situ oxidative polymerization of PANI, Long et al. [117] acquired a PANI/Mg-Al layered double oxide (PANI/LDOs). SEM and TEM data disclosed that PANI/LDOs presented unique nanoflower–nanofiber hybrid structures with very rough surfaces, where relatively aggregated spherical PANI was evenly dispersed on the LDO surface. Meanwhile, it possessed a high S_BET_ of 49.23 m^2^ g^−1^, a large V_pore_ of 0.31 cm^3^ g^−1^, and high N content. When taken for Cr(VI) capture, it realized a maximum adsorption capacity of 409.77 mg g^−1^ in a 100 mg L^−1^ solution at pH = 2. Mechanistic research disclosed that the adsorption mechanisms were mainly dominated by strong electrostatic attraction and surface complexation. Furthermore, to simultaneously achieve the adsorption and degradation of Cr(VI), Sun et al. [92] manufactured a sandwich-structured PANI/MXene/GO (PMG) with obvious layer spacing, a high S_BET_ of 15 m^2^ g^−1^, and a V_pore_ of 0.076 cm^3^ g^−1^. Based on the large interlayer spacing-induced highly exposed active sites and rapid diffusion channels, PMG realized 89.3% of Cr(VI) transformation into Cr(III) in a 160 mg L^−1^ solution. Notably, the adsorption coupled with degradation was mainly due to electrostatic interaction, redox reactions, and complexation.

To build self-supporting adsorbents, Liang et al. [104] designed a PANI@SA-SNM gel by hybridizing MXene, PANI, and sodium alginate (SA). SEM indicated that the PANI@SA-SNM gel held dense microporous network structures, where wrinkles and granules were staggered, thus exposing more contactable surface. Meanwhile, it acquired an S_BET_ of 5.7 m^2^ g^−1^, an average pore size of 1.9 nm, and copious -NH, -OH, and -COOH groups. Adsorption experiments disclosed that it exhibited an excellent capacity stability across a wide pH range of 2–7, which was mainly because of its chemisorption-dominated adsorption behavior. As a result, it obtained maximum adsorption capacities of 255.81 mg g^−1^ for Cu(II) and 352.76 mg g^−1^ for Hg(II) in a 1000 mg L^−1^ solution at pH = 4, respectively. Notably, it still presented >90% initial adsorption capacities for both ions after eight adsorption–desorption cycles. Mechanistic research disclosed that the adsorption mechanism includes physical adsorption, ion exchange, electrostatic attraction, complexation, and redox reaction.

#### 5.1.2. Organic Pollutants

Typical organic pollutants in water systems include pharmaceuticals, hormones, pesticides, dyes, and fire suppressants. What is worrying is that industrial and domestic wastewater discharge 300 million tons of micro-pollutants each year. The primary sources involve the pharmaceutical industry, medical waste, agricultural activities, and personal care products [129,130]. Notably, these organic pollutants are persistent and poorly biodegradable, making them extremely harmful to the ecosystem and the human body [131]. Compared with other removal techniques, the adsorption method is favored for its advantages of simple operation, high efficiency, low-cost, good sustainability, and excellent reusability [132].

For easy adsorption of Rhodamine B (RhB), Varghese et al. [123] constructed a ternary PEG-capped PANI/TiO_2_/CuO with high dispersion, an excellent S_BET_ of 55.5 m^2^ g^−1^, a large V_pore_ of 0.12 cm^3^ g^−1^, and a small particle size. When used for the adsorption of 5 mg L^−1^ RhB, it acquired a splendid capture capacity of 3.53 mg g^−1^ and only showed <10% capacity drop after five adsorption–desorption cycles. Based on the joint action of PANI, Jia et al. [23] fabricated a novel ACNF/PANI/MIL-101(Cr)-NH_2_. Therein, the presence of ACNF realized the uniform growth of PANI particles, while the presence of PANI ensured the effective recombination of MIL-101(Cr)-NH_2_. Thus, ACNF/PANI/MIL-101(Cr)-NH_2_ presented a substantial 3D-supporting framework and tertiary porous structure with a high S_BET_ of 359.2 m^2^ g^−1^, a large V_pore_ of 0.28 cm^3^ g^−1^, and rich N-containing functional groups. When utilized for indomethacin (IDM) capture, it realized an outstanding adsorption capacity of 400.1 mg g^−1^ in 400 mg L^−1^ IDM, and the rebuilt self-supported ACNF/PANI/MIL-101(Cr)-NH_2_ only presented 10.98% capacity drop after five adsorption cycles. Comprehensive analysis manifested that the adsorption mechanism was primarily due to pore-filling, electrostatic interaction, hydrogen bonding, and π-π interaction.

To alleviate organic pollution in actual water, Wang et al. [133] constructed a unique PANI@PCDP via loading PANI on porous β-cyclodextrin (PCDP). SEM data depicted that coral-like structural PANI were present not only on the surface of PCOP but also internally. As a result, PCDP offered 3D robust porous frameworks and inhibited PANI agglomeration, while PANI provided copious =N- and -NH_2_ groups and obvious positive charges for PANI@PCDP (point of zero charge at pH 8.8). Consequently, it delivered a nearly 100% removal rate for bisphenol A (BPA) in a mixed BPA (22.8 mg L^−1^) and Cr(IV) (50 mg L^−1^). Moreover, it could also adsorb Cr (IV) (98% removal) and then degrade to produce low-toxicity Cr(III). In another research, Mao et al. [122] manufactured an easy-to-recycle core–shell structural cotton fiber-anchored PANI@LDH (CF@PANI@LDH). SEM indicated that nanowire-like PANI was staggeredly dispersed on the CF surface, building a 3D staggered structure with many gaps. Meanwhile, layered structural LDH was evenly fixed on the CF@PANI surface. As a result, the unique self-supported core–shell structure effectively exposed accessible active sites of PANI and LDH. When adopted for ketoprofen removal, it acquired an excellent capture capacity of 588.24 mg g^−1^ in a 500 mg L^−1^ solution. Mechanism analysis disclosed that the adsorption mechanism was attributed to hydrogen bonding and π-π interaction between PANI and ketoprofen, and ion exchange between LDH and ketoprofen.

#### 5.1.3. Harmful Gas

Currently, the harmful gases that need to be dealt with urgently include CO_2_, SO_2_, and VOCs [134]. The primary sources involve unreasonable resource exploitation, massive chemical fuel combustion, hazardous chemical reagent use, and daily industrial and life activities [135]. These emissions are the producers of environmental pollutants such as greenhouse gases and acid rain, and in some situations may cause serious harm to human health [136]. The adsorption method is an effective method for gas separation and enrichment, but low-performance adsorbents are still the key factor restricting their adsorption performance.

As is well known, constructing porous PANI nanosheets for gas adsorption faces a huge challenge. Innovatively, Zhang et al. [137] prepared a micropore-dominated ultrathin PANI nanosheet from a hexaaminobenzene monomer by a LiCl/KCl molten salt-assisted method. With an ultrathin thickness of 1.6 nm and a high S_BET_ of 395 m^2^ g^−1^, this ultrathin porous PANI nanosheet illustrated an outstanding CO_2_ adsorption capacity of 1.93 mmol g^−1^ at 25 °C and 1.0 bar, which was far higher than that of un-modified PANI (mmol g^−1^). To highly expose the active sites of PANI, Yoo and colleagues [138] designed a unique PANI@MIL-101(Cr) based on a bottle strategy in which PANI was evenly distributed on the surface of MIL-101(Cr). Research indicated that MIL-101(Cr) with reasonable PANI loading showed a clearly increased CO_2_ adsorption capacity of 1.7 mmol g^−1^ even at a low pressure of 0.15 bar at 25 °C, whereas single MIL-101(Cr) only acquired a low capture capacity of 0.7 mmol g^−1^. Notably, it maintained almost 100% capacity retention after six adsorption–desorption cycles. Mechanism analysis showed that the design of PNAI with a reasonable size and distribution can effectively inhibit its pore-blocking effect on MIL-101(Cr).

### 5.2. Capacitive Deionization

CDI, also called electrosorption, is a promising technique for the disposal of seawater or brackish water that depends on the principle of EDL capacitors (EDLCs) [139]. PANI-based composites have delivered huge potential for CDI applied in wide water treatment applications (Table 2). The positive surface charge, rich amino and imino groups, and excellent conductivity of PANI guarantee the selective capture of diverse anions. PANI modified by functional groups or transition metals can achieve selective capture of toxic heavy metal or rare metal ions in industrial wastewater. Spinel-modified PANI can be utilized for the selective extraction of lithium. Advanced PANI-CDI systems with flow electrode structures and even membrane introduction can be used for lasting wastewater purification. Moreover, PANI-based current collectors provide a solid possibility for the improvement and stability of FCDI performance. As capacity, selectivity, stability, and regenerability continue to optimize, PANI-based composites hold promise for ensuring efficient CDI treatment across a wide range of water sources.

#### 5.2.1. Desalination

Seawater desalination is a critical approach to alleviating global water scarcity, particularly in arid, semi-arid, and island regions. However, traditional methods like electrodialysis, reverse osmosis, and forward osmosis are energy-intensive and tend to produce hazardous byproducts [157,158]. In contrast, CDI purifies water by imposing a low potential to the anode and cathode to capture ions, and this process is highly reversible [159]. This renders it a promising and sustainable option for seawater desalination. Additionally, CDI delivers favorable compactness and mobility along with low maintenance requirements, which also meets the urgent requirements of remote areas or mobile platforms [160]. Consequently, it provides sustainable and energy-saving strategies to accelerate the alleviation of the global water crisis.

Ma et al. [140] designed a hollow hierarchical tubular cobalt hexacyanoferrate/PANI (CoHCF/PANI). Viewing the synergistic effect between uniformly dispersed CoHCF and 3D interconnected PANI tubes, it acquired an excellent S_BET_ of 200.55 m^2^ g^−1^, a large V_pore_ of 0.17 cm^3^ g^−1^, and superior specific capacitance of 263.8 F/g. CDI tests depicted that CoHCF/PANI delivered an excellent desalination capacity and rate of 30.48 mg g^−1^ and 3.66 mg g^−1^ min^−1^ in 500 mg L^−1^ NaCl at 1.2 V, and it only displayed a 1.9% capacity drop after 50 CDI cycles. To construct self-supporting electrodes, Tian et al. [47] fabricated an activated carbon fiber (ACF)/PANI (ACF/PANI). SEM data showed that after electrochemical polymerization, the smooth ACF surface was covered with evenly distributed, thin, and dense PANI layers, thus inducing massive defect sites and rapid desalination dynamics. CDI desalination results indicated that ACF/PANI obtained a high desalination capacity of 19.9 mg g^−1^ under a high mass loading of 8.67 mg/cm^2^ in 200 mg L^−1^ NaCl at 2.0 V. Notably, it also realized an ultrahigh capacity retention of nearly 100% even after 28 CDI cycles. Innovatively, Li et al. [139] prepared a sandwich-structured GO/PANI/PBA (GO/PANI/PBA) with large layer spacing, superior S_BET_ of 148.08 m^2^ g^−1^, and a large V_pore_ of 0.66 cm^3^ g^−1^. As a result, it not only delivered an ultrahigh desalination capacity of 91.6 mg g^−1^ in 500 mg L^−1^ NaCl at 1 A g^−1^, but also realized an excellent water disinfection ability with 94.0% ± 3.1% Escherichia coli inactivation efficiency.

To achieve a continuous desalination process, Luo et al. [161] proposed a novel approach and designed a rod-like PANI/AC (PAC) composite flow electrode. SEM displayed that PANI was not only loaded on the AC surface, but also grew between adjacent ACs, thus constructing a 3D framework structure with continuous conductivity and rich pores. This structure greatly reinforced the stability of PANI and the exposure of active sites. Thus, the optimal PAC-9 acquired both an ultrahigh salt removal efficiency of 94.3% and a rapid average salt removal rate of 0.963 μmol/cm^2^/min in 2000 mg L^−1^ NaCl at 1.2 V. Notably, it realized nearly 100% capacity retention even after 12 h of lasting operation. Further, Chen et al. [162] adopted porous PANI (p-PANI) to replace common PANI, building a similar 3D continuous framework structural p-PANI/AC with a higher S_BET_ of 616.1 m^2^ g^−1^ than that of PANI/AC (213.5 m^2^ g^−1^). This structure favored exposing more accessible sites, enhancing conductivity, and reducing the flow electrode viscosity. Innovatively, from the perspective of current collectors, Chen et al. [48] took PANI/graphite (GP) channels to replace the typical GP channels, further optimizing the insufficient conductive route. Thus, the average salt removal rate of PGP-FCDI in 2000 mg L^−1^ NaCl at 1.2 V was increased by 1.46-fold, while electrode sedimentation was decreased by 15%.

#### 5.2.2. Toxic Anion Adsorption

Presently, the content and types of toxic anions in water are increasing. Chlorine can reduce the biodiversity of aquatic organisms and even local organisms, and severely corrode industrial and life pipelines [147]. Nitrates can induce eutrophication of water bodies and convert them into carcinogenic nitrite in human bodies [148]. Sulfate can deteriorate water quality and cause human diarrhea at high concentrations [163]. Fluoride can cause dental fluorosis and skeletal fluorosis in humans, and high fluoride contents disrupt the metabolic processes of aquatic organisms like fish and algae, destroying the water ecological balance [93]. The removal of such ions is very difficult, and electro-driven CDI technology offers a suitable strategy [149]. Currently, there is extensive research on chloride-selective adsorption, and the selective adsorption of other anions needs to be strengthened.

Liang et al. [150] prepared a PANI and TiO_2_ co-modified ACF (PANI/TiO_2_/ACF) for Cl^−^ capture. SEM indicated that TiO_2_ nanoparticles with sizes of 100–200 nm was homogeneously dispersed on the surface of the smooth ACF with diameters of 9 μm. Meanwhile, PANI/TiO_2_/ACF also integrated excellent hydrophilicity and pseudocapacitance from TiO_2_ and significantly improved conductivity from PANI. As a result, it delivered a high Cl^−^ adsorption capacity of 17.1 mg g^−1^ in 200 mg L^−1^ NaCl at 2.0 V, which was 1.4 times that of the single ACF. Fateminia et al. [148] exploited a densely spaced network structural AC/PVDF/PANI/ZrO_2_ for NO_3_^−^ removal. Thereinto, single PANI could enhance the composite conductivity, while single ZrO_2_ realized a 19.43% specific capacitance rise and a 34.27% charge transfer resistance drop. Notably, PANI coupled with ZrO_2_ acquired a 75.13% ultrahigh specific capacitance rise and a 55.47% charge transfer resistance drop. When used for NO_3_^−^ capture, it obtained a high desalination capacity of 6.01 mg g^−1^ in 70 mg NaNO_3_ at 1.2 V. In the aspect of fluoride adsorption, Lu et al. [93] designed a continuous conductive structural MS-Ti_3_C_2_T_x_ MXene/PANI/AC. Viewing the bridging effect of PANI, MXene, and AC were tightly packed, which not only improved the contact efficiency between the electrode and electrolyte but also achieved charge transfer in multiple ways. Thus, it delivered an ultrarapid average removal rate of 3.415 mg m^−2^ s^−1^ in 10 mg L^−1^ NaF solution at 1.2 V, which was 1.72 times higher than that of single AC. More importantly, it still kept >85% initial capacity after 50 CDI cycles. Further research disclosed that the high defluorination ability was because of the electrostatic interaction, ion intercalation, redox reactions, and ligand exchange with terminal groups.

#### 5.2.3. Heavy Metal Ion Capture

As mentioned in Section 5.1.1, heavy metal ions are highly toxic and pose enormous risks to the environment and human health. Thus, it is crucial to realize effective remission and control the pollution. This urgency has prompted researchers to explore CDI technology, which has already proven successful in seawater desalination. Recent research on the CDI application for heavy metal capture illustrates that it is a promising solution [164]. Although early results are encouraging, further research is needed to optimize CDI techniques for efficient heavy metal removal and to evaluate their effectiveness.

Bharath et al. [165] fabricated a PANI-modified date seed-derived AC (PANI-DSAC). SEM data disclosed that single DSAC delivered porosity-developed quasi-cube structure, and PANI with short fibrous structures was interlaced and uniformly covered on the DSACs. Thus, the definitive PANI-DSAC exhibited a much more accessible surface with a splendid S_BET_ of 928 m^2^ g^−1^ and a pore size of 8 nm for capturing Fe^2+^ and Cu^2+^ from mining wastewater. Eventually, it realized a maximum Fe^2+^ removal efficiency of 99.2% at 20 mg L^−1^ Fe^2+^ containing 0.05 M H_2_SO_4_ at 1.2 V. Notably, compared with Fe (II), Cu (II) is easier to adsorb in mixed simulated wastewater. Mechanistic analysis revealed that the amino and imino groups of PANI possessed a higher affinity for Cu(II). In another research, Feng et al. [151] built a PA-doped PANI/PC (PA-PANI/PC) carbon cloth electrode with rough fiber surfaces and proposed a novel approach of coupling CDI with deposition for In(III) extraction. The optimized PA-PANI/PC-2 delivered a maximum In(III) extraction capacity of 284.67 mg g^−1^ in 400 mg L^−1^. In(III) at 1.2 V and pH = 2.5. Notably, it presented only a 22% capacity drop after seven CDI cycles. Further research indicated that the high recycling efficiency was because of the strong complexation ability of PO_4_^3−^ with In(III).

#### 5.2.4. Water Softening

Water hardness, raised by mineral ions involving Ca^2+^ and Mg^2+^, remains a common issue across industrial and domestic environments [166]. These ions tend to produce insoluble precipitates such as CaCO_3_ and MgCO_3_ [167]. This can adversely affect the service life of household appliances and the health of skin and hair. Orthodox water softening techniques, however, often consume massive energy or require large amounts chemical reagents [168]. Presently, CDI is seen as a promising technique for water softening. Advancements in CDI-based water softening not only address key limitations of traditional methods but also offer substantial potential for advancing sustainability in water treatment. To effectively remove Ca(II) and Mg(II), Tan et al. [152] fabricated a microalgae-derived hierarchical porous carbon (HPC) and then synthesized MnO/HPC and PANI/HPC. Notably, both MnO/HPC and PANI/HPC had similar morphologies, where MnO nanorods was evenly dispersed on the surface of 3D HPC, and PANI formed a nanofiber@3D HPC structure. When assembled as MnO/HPC-PANI/HPC systems, they realized high desalting capacities of 0.65, 0.71, and 0.76 mmol/g for NaCl, MgCl_2_, and CaCl_2_, respectively. Thereinto, MnO/HPC delivered a selectivity order of Ca ≥ Mg > Na, mainly owing to the stronger binding strength in the MnO cavity. Multiscale simulations disclosed that MnO/HPC with the unique luminal structure of MnO and HPC could produce superior intercalation selectivity for divalent cations.

#### 5.2.5. Resource Recovery

CDI techniques have manifested excellent application potential in the selective extraction and recovery of high-value elements, involving nitrogen, phosphorus, lithium, potassium, and uranium from wastewater or brackish water [28,156]. Notably, CDI is well-suited for the separation of nitrogen and phosphorus from wastewater [153]. These recovered elements can be further processed into fertilizers for agricultural applications. Meanwhile, the application of CDI in salt-lake lithium extraction and nuclide enrichment has gradually shown strong applicability [156]. The utilization of CDI in resource recovery greatly contributes to advancing the circular economy while reducing the environmental impacts associated with traditional resource mining.

δ-MnO_2_ is an excellent material for NH_4_^+^ capture, but it suffers from serious layered self-aggregation. Wang et al. [153] built a PANI-intercalated MnO_2_/CNTs (P/M-C-X) with a high S_BET_ of 221.4 m^2^ g^−1^ and a large V_pore_ of 0.27 cm^3^ g^−1^ via a rapid inorganic/organic interface reaction. Notably, PANI intercalation augmented the layer spacing and O vacancies of δ-MnO_2_, and the introduction of CNTs further supported and stabilized the 3D structures while promoting mass/charge transfer dynamics. Viewing the unique 1D/2D structure, P/M-C-50 obtained a splendid NH_4_^+^ adsorption capacity and rate of 160.0 mg g^−1^ and 1.07 mg g^−1^ s^−1^ in 400 mg L^−1^ NH_4_^+^ at 1.4 V. To effectively realize U(VI) enrichment, Shuang et al. [27] fabricated a self-supporting CNT/CA/PANI (CCP). SEM displayed that massive dispersed and continuous CNTs with diameters of 20–30 nm was interlaced with PANI nanofibers, thus forming a 3D stable porous framework. Also, it possessed a superior S_BET_ of 687 m^2^ g^−1^, a large V_pore_ of 0.28 cm^3^ g^−1^, and a reasonable pore-size distribution of 3.5 nm by a facile electrodeposition craft. The well-built structure delivered the following two core advantages: (i) multiplied active sites of synergistic -NH- of PANI and -OH of CS enhanced the selective capture of U(VI); and (ii) optimized hierarchical structures and surface wettability accelerated the desalination kinetics of U(VI) and alleviated the co-ion effect. Eventually, viewing EDL coupling with pseudocapacitance and UO_2_^2+^⋯ NH specific binding, CCP realized a superior U(VI) adsorption capacity of 344.8 mg g^−1^ in 100 mg L^−1^ U(VI) at 0.9 V and pH 4.5. Moreover, it still reached 85% capacity retention after seven CDI cycles. Li et al. [169] designed NPHPC/nano-TiO_2_/PANI (STP) via rapid electrodeposition and delivered a splendid adsorption capacity of 307.25 mg g^−1^ in 100 mg L^−1^ U(VI) at 0.9 V and pH 4.5. Mechanism analysis showed that rich N and P heteroatoms in NPHPC offered additional pseudocapacitance, while homogeneously dispersed PANI provided high-affinity functional groups for U(VI). As a core application potential, by compounding with specific materials, PANI-based composites have a high selectivity for multifarious ions. For instance, Zhang et al. prepared a ZnFe-LDH-PANI/CNT heterojunction for phosphorus removal [28], and Zhan et al. [170] fabricated PVA-PANI-H_1.6_Mn_1.6_O_4_ for lithium extraction.

## 6. Regeneration Utilization Strategy

### 6.1. Non-Electrochemical Adsorption

On the application side, the non-electrochemical adsorption application mainly refers to adsorption. Currently, the regeneration technology of PANI-based composites is primarily the washing method, and the detergents adopted mainly involve acidic solutions, alkaline solutions, alcohol reagents, and other reagents [171,172]. Thereinto, detergents frequently used were methanol and ethanol because of their wide applicability and excellent performance [173]. Normally, the concentration range of the solution treated by adsorption is large, involving both high concentration (e.g., >200 mg/L) and low concentration (e.g., <50 mg/g). In addition, the selection of detergents influences the adsorption performance of the recovered material. For HPCNs, their adsorption behavior involves both physical and chemical effects, such as the pore-filling effect, electrostatic interaction, hydrogen bonding, hydrophilic/hydrophobic interaction, and π-π interaction. Bai et al. [174] prepared PANI-based cellulose-sodium alginate composite (PNC) gel via electron beam radiation for the adsorption of MB. After the adsorption is completed, the desorption operation is carried out with 1 mol/L HCl solution as the eluent to regenerate the adsorbent. It was found that the removal rate of MB was still as high as 90% after five adsorption–desorption cycles. For most of the adsorption cycle experiments, the number of adsorption cycles set by the researchers is usually five, and the step is usually laborious. The combination of adsorption–degradation technology or the use of electrosorption instead of traditional adsorption may be a potential research direction.

### 6.2. Electrochemical Adsorption

On the application side, the electrochemical adsorption application mainly refers to CDI. For PANI-based composites, the binding mechanism of composites and ions (e.g., Li^+^, Na^+^, K^+^, Cu^2+^, Pb^2+^, F^−^, Cl^−^, NO_3_^−^, and SO_4_^2−^) is mainly electrostatic interaction, ligand exchange, and surface complexation, which is a physical–chemical adsorption process. In the adsorption stage, by applying a low voltage (<2.0 V) at both ends of the electrodes, the anions and cations from the salt solution can rapidly migrate and adsorb on the opposite electrode surface [175]. Analogously, in the desorption stage, through reverse operation (such as 0.8 V), adsorbed ions on the electrode surface are desorbed and returned to the solution, thereby realizing the regeneration and utilization of the electrode [176]. Notably, for the whole desorption process, acidic solutions, alkaline solutions, and alcohol reagents are not introduced, which strongly reflects the concept of cost reduction and green chemistry.

## 7. Challenges and Opportunities

Although there have been widespread investigations on developing advanced PANI-based composites for high-performance electrochemical and non-electrochemical adsorption applications, further research is required to sufficiently understand the drawbacks of PANI-based composites and how to further effectively reinforce them to maximize the material performance.

(i) Current PANI-based composites often undergo challenges such as uneven interface bonding (e.g., weak physical interaction between PANI and MOFs) or blocked active sites, which simultaneously restrict traditional adsorption ability and CDI adsorption kinetics. Future research should localize site-specific interface regulation. Specifically, ligand-directed in situ polymerization should be developed to graft PANI chains onto the functional groups of supports (e.g., -OH on LDHs, -COOH on MXene) via covalent bonds or coordination bonds, optimizing PANI dispersion to avoid pore blockage of PCs/MOFs (key for traditional adsorption) while building continuous conductive pathways (key for CDI). Moreover, advanced characterization techniques (e.g., operando X-ray photoelectron spectroscopy, in situ Raman spectroscopy) should be integrated to monitor the dynamic evolution of the PANI-support interface under adsorption equilibrium (traditional adsorption) and electric field (CDI) conditions, elucidating how interface interactions affect pollutant coordination and ion intercalation, thus guiding the design of high-performance composite interfaces.

(ii) In the future, it is vital to develop magnetic or self-supporting PANI-based composites to realize their effective performance release and rapid material recovery. For non-electrochemical applications, PANI-based composites have a strong ability; however, a tedious recovery step is often required, especially for particles with small sizes and low density. This greatly adds to the overall treatment cost. Thus, introducing magnetic particles into PANI-based composites is necessary, which can not only solve the recycling issues but also improve the reusability of OAPCs. To meet all applications, constructing self-supported PANI-based composites is more strongly advocated. This is similar to ACF and activated carbon cloth, which contain 100% active material and copious porosity. Currently, constructing carbon aerogel-based@PANI composites is a vital research direction.

(iii) It is vital to explore the effective integration of traditional adsorption and advanced CDI desalination in emerging fields. Specifically, switchable PANI-based composites should be developed to realize mode conversion between adsorption and CDI via pH or voltage adjustment. Under neutral conditions, PANI’s protonated amino groups can be used to adsorb organic pollutants via hydrogen bonding (traditional adsorption). Under applied voltage (1.0–1.6 V), CDI systems can be activated to remove salts and heavy metal ions, meeting the variable treatment needs of different water sources. For gas–water dual treatment, PANI-based composites can be extended to simultaneous VOC adsorption and heavy metal ion capture, adopting PANI’s tunable porosity and functional groups to achieve multi-phase pollutant capture. More importantly, coupling adsorption technology with other technologies (e.g., supercapacitors, battery, photocatalysis, and electro-Fenton) to realize self-powered operation, along with full adsorption and high degradation of pollutants, is another vital research direction.

(iv) Currently, artificial intelligence has been extensively applied in pharmaceutical research. As one subset, machine learning (ML) enables computer software to predict outcomes without the need for massive programming. A key characteristic of ML lies in its capability to train algorithms for handling complex and large-scale multidimensional datasets. Although existing studies have proved the application of ML in PANI-based composites for environmental remediation, this field remains in the early development stage. ML-based prediction models hold the potential to greatly reduce research workloads, making it worthwhile to explore the comprehensive application of ML in composite material combination prediction and production condition prediction.

## 8. Conclusions

Overall, due to the superior mass–charge transfer ability, tunable morphology, copious N-containing functional groups, and high structural tunability, PANI-based composites obtain huge research attraction. This review systematically summarizes the design principles, composite construction strategies, and adsorption applications of PANI-based composites. Key design principles, including micro-support skeleton construction, conductive skeleton introduction, and selective active site anchoring, are proposed. These principles aim to address defects of single components and realize the improvement of the properties, selectivity, and stability of PANI-based composites. Subsequently, multiple composites, e.g., PANI/porous carbon, PANI/metal oxide, PANI/MXene, PANI/MOF, PANI/LDH, and PANI/PBAs are analyzed. Eventually, their applications in electrochemical and non-electrochemical adsorption are comprehensively assessed, involving desalination, toxic anion/heavy metal ion removal, water softening, resource recovery, and organic pollutant and harmful gas adsorption. Based on the above review, we think that the future research direction of PANI-based composites mainly focuses on the controllable synthesis of magnetic or self-supporting composites at the molecular-level and at low time–cost. Meanwhile, cross-application approaches can be used to achieve self-powered operation (facing CDI), along with full adsorption and high degradation of pollutants.

## Figures and Tables

**Table 1:** Comprehensive comparison of PANI-based composites in diverse pollution adsorption.

Adsorbents	Pollutants	Solution (mg L^−1^)	pH	Dosage: m/V (mg mL^−1^)	q_e_ (mg g^−1^)	Ref.
PANI/Mg/Al-LDH	Cr(VI)	40–320	1.0–10.0	5/50	156.2	[97]
Fe-MOF@PANI	Cd(II)	25–150	3.0–8.0	5/50	143.36	[98]
Fe-MOF@PANI	Pb(II)	25–150	3.0–8.0	5/50	258.59	[98]
PANI@SA-SNM gel	Hg(II)	50–1000	2.0–7.0	20/50	352.76	[104]
PANI@SA-SNM gel	Cu(II)	50–1000	2.0–7.0	20/50	255.81	[104]
PANI-Co CNRs	Pb(II)	50–150	2.0–6.0	10/50	ca. 750	[115]
CMC/GO/PANI	Cu(II)	2.5–500	2.0–5.5	20/20	247.69	[116]
PANI/LDOs	Cr(VI)	50	2.0–10.0	10/100	409.77	[117]
BC@PANI	Cr(VI)	100–1000	2.0–10.0	15/20	877.19	[18]
Ppy-PA-Pani	Cr(VI)	10–200	3.0	10/50	222.72	[118]
PANI-Co CNRs	Pb(II)	50–150	2.0–6.0	10/50	ca. 750	[115]
ACNF/PANI/MIL-101(Cr)-NH_2_	IDM	50–400	5.0–11.0	20/50	450	[23]
ACNF/PANI	IDM	50–400	5.0–11.0	20/50	194.3	[23]
ZC@PANI@NiAl-LDH	Saccharin	50–500	3.0–10/0	20/40	498.5	[27]
CS@PANI@ZnAl-LDH	Naproxen	50–400	4.5–10.0	20/40	545.5	[64]
DESs-PANI@FeO	MO	50–400	4.0–10.0	10/50	812	[77]
DESs-PANI@FeO	MB	50–400	4.0–10.0	10/50	446	[77]
CS@PANI@LDH	DCF	100–500	5.5–10	20/40	618.16	[105]
PAIS	MB	25–100	2.0–13.0	100/25	454.54	[119]
PANI-MnPBA/NiCoMnS	CR	15–45	3.0–11.5	_	252.2	[120]
MCC/COF/PANI	IDM	10–100	nature	2/10	149.1	[121]
CF@PANI@LDH	Ketoprofen	100–500	4.5–10.0	15/80	588.24	[122]
PANI/TiO_2_/CuO	RhB	5–20	nature	10/50	3.53	[123]

**Table 2:** Comprehensive comparison of PANI-based composites in diverse CDI application fields.

Electrodes	Application Fields	Solution (mg L^−1^)	Voltage (V)	SAC (mg g^−1^)	Refs.
PANI/Mn_2_O_3_	Desalination	500	1.2	ca. 13.0	[26]
PAC/PANI	Desalination	500	1.2	35.3	[66]
Ag@PANI/AC	Desalination	500	1.2	19.2	[73]
CoHCF/PANI	Desalination	500	1.2	23.07	[140]
ACF/PANI	Desalination	200	2.0	19.9	[47]
SiW_12_@PANI/EGC	Desalination	500	1.2	23.1	[141]
GO/PANI/PBA	Desalination	500	1.2	ca. 81.0	[139]
PANI-AC	Desalination	500	1.0	14.7	[142]
MXene/PANI/Acid-MWCNTs	Desalination	500	1.2	95.04	[143]
Ti_3_C_2_T_x_/PANI/PPY	Desalination	500	1.2	39.62	[144]
S-Ti_3_C_2_T_x_/PANI/F-Ti_3_C_2_T_x_	Desalination	500	1.2	77.38	[145]
M93A	Desalination	500	1.2	50.6	[146]
PANI@PG	Toxic anion adsorption	500	1.2	ca. 35	[147]
AC/PVDF/ZrO_2_	Toxic anion adsorption	70	2.0	6.01	[148]
PACP	Toxic anion adsorption	585	1.2	65	[149]
ACF/TiO_2_/PANI	Toxic anion adsorption	254	17.1	2.0	[150]
PA-PANI/PC-2	Heavy metal ion capture	400	1.2	284.67	[151]
PANI/HPC	Water softening	555	1.2	84.36	[152]
PANI/HPC	Water softening	475	1.2	67.45	[152]
LDH@MOF/PANI	Resource recovery	6	1.2	5.6	[29]
CC/PAN	Resource recovery	100	0.9	223	[51]
P/M-C-50	Resource recovery	400	1.5	160.0	[153]
ZnFe-PANI/CNT	Resource recovery	6	1.2	12	[154]
CCP-2	Resource recovery	100	0.9	344.8	[27]
Fc-PANI/CNTs//PANI/CNTs	Resource recovery	300	1.2	35	[155]
GO/α-MnO_2_/PANI	Resource recovery	100	0.9	330.41	[156]

## Data Availability

No new data were created or analyzed in this study. Data sharing is not applicable to this article.
